# Study Protocol of a Prospective, Monocentric, Single-Arm Study Investigating the Correlation of Endograft Properties with Aortic Stiffness in Abdominal Aortic Aneurysm Patients Subjected to Endovascular Aortic Repair

**DOI:** 10.3390/jcm13082205

**Published:** 2024-04-11

**Authors:** Manolis Abatzis-Papadopoulos, Konstantinos Tigkiropoulos, Spyridon Nikas, Katerina Sidiropoulou, Christina Alexou, Kyriakos Stavridis, Dimitrios Karamanos, Vasilios Kotsis, Ioannis Lazaridis, Nikolaos Saratzis

**Affiliations:** 1 Vascular Unit, 1st University Surgical Department, Papageorgiou General Hospital, Aristotle University of Thessaloniki, 56403 Thessaloniki, Greece; kostastig@yahoo.com (K.T.); kasidi94@hotmail.com (K.S.); kstavridis17@yahoo.gr (K.S.); dkaramanos@gmail.com (D.K.); drlazaridis@yahoo.gr (I.L.); nicos_saratzis@yahoo.com (N.S.); 2 Radiology Department, Papageorgiou General Hospital, 56403 Thessaloniki, Greece; sncorfu@hotmail.com; 3 Cardiothoracic Surgery Department, Papanikolaou General Hospital, 57010 Thessaloniki, Greece; christinaalexou@gmail.com; 4 3rd University Department of Internal Medicine, Papageorgiou General Hospital, Aristotle University of Thessaloniki, 56403 Thessaloniki, Greece; vkotsis@auth.gr

**Keywords:** abdominal aortic aneurysm, pulse wave velocity, aortic compliance, aortic stiffness, endovascular aortic repair, aortic endograft

## Abstract

The number of endovascular aortic repairs (EVARs) has surpassed the number of open surgical repairs of abdominal aortic aneurysms (AAAs) worldwide. The available commercial endoprostheses are composed of materials that are stiffer than the native aortic wall. As a consequence, the implantation of stent–graft endoprostheses during EVAR increases aortic rigidity and thus aortic stiffness, resulting in a decrease in abdominal aorta compliance. EVAR has been found to have a possibly harmful effect not only on heart functions but also on other vascular beds, including kidney function, due to the decrease in aortic compliance that it causes. Aortic stiffness is measured by various hemodynamic indices like the pulse wave velocity (PWV), the central aortic pressure (CAP), and the augmentation index (AIx). In the literature, there are increasing numbers of studies investigating the properties of endografts, which are strongly related to increases in aortic stiffness. However, there is a lack of data on whether there is a correlation between the length of various endografts implanted during EVAR and the increase in the PWV, CAP, and AIx postoperatively compared to the preoperative values. The aim of this prospective, observational, monocentric, single-arm study is to investigate the correlation between endograft length and the postoperative increase in the PWV, CAP, and AIx in patients subjected to EVAR. Additionally, this study intends to identify other endograft properties related to increases in the PWV, CAP, and AIx. Other endpoints to be studied are the existence of immediate postoperative myocardial and kidney injury after EVAR. The prediction of cardiovascular events caused by endograft-related increased aortic stiffness could contribute to the improvement of various endograft properties so that the impact of endografts on the native aortic wall can be minimized.

## 1. Aortic Stiffness and Aortic Compliance

Before the 1990s, open repair was the only treatment option for abdominal aortic aneurysms (AAAs) [1]. The first deployment of a stent–graft device in a person was conducted by the Ukrainian surgeon N. Volodos, who performed endoluminal treatment of a traumatic thoracic aneurysm using a “homemade” stent–graft device. Endovascular aortic repair (EVAR) of AAAs was introduced by Parodi et al. in 1991 [2]. The number of EVARs has now surpassed the number of open surgical repairs of AAAs worldwide [3]. This is partially attributed to the low morbidity and mortality associated with EVAR during the immediate postoperative period compared to the open surgical repair of AAAs, especially in elderly patients and patients with ruptured AAAs, whose first treatment option for AAAs should be EVAR, according to the European Society of Vascular Surgery (ESVS) guidelines [4].

A great variety of endografts with different properties, produced by various companies, are available to health professionals [5]. The available commercial endoprostheses are composed of either Dacron polyester or expanded polytetrafluoroethylene (ePTFE) supported by a skeleton, which is, in the majority of cases, a stent composed of nitinol or other metal alloys. As a consequence, the implantation of stent–graft endoprostheses during EVAR increases the aortic rigidity and, thus, the aortic stiffness, resulting in a decrease in abdominal aorta compliance [6]. Aortic stiffness has been established as an independent factor for increased cardiovascular disease risk [7].

The classic definition of compliance is the change in blood volume relative to a given change in distending pressure [8]. Along the aorta, ascending to its abdominal part, its elasticity and, respectively, its compliance decrease [9]. The direct measurement of regional aortic compliance is difficult because there is no simple means of estimating regional changes in blood volume [8]. However, there are various hemodynamic indices for measuring aortic stiffness like the measurement of the aortic pulse wave velocity (PWV), the central aortic pressure (CAP), and the augmentation index (AIx) [10].

## 2. Pulse Wave Velocity (PWV)

**Figure 1:**
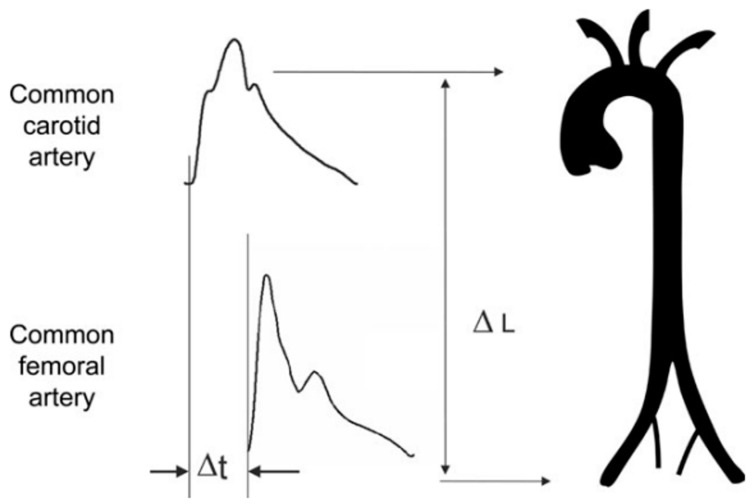
Carotid–femoral pulse wave velocity measurement using the foot-to-foot method. ΔL: distance; Δt: transit time [10].

The measurement of the PWV is generally accepted as the most simple, non-invasive, robust, and reproducible method for determining arterial stiffness. The carotid–femoral PWV is a direct measurement, and it corresponds to the widely accepted propagative model of the arterial system. The PWV is usually measured using the foot-to-foot velocity method from various waveforms. These are usually obtained transcutaneously at the right common carotid artery and the right femoral artery, hence the term ‘carotid–femoral’ PWV, and the time delay or transit time (defined as Δt, measured in seconds) is measured between the feet of the two waveforms. The distance (defined as L, measured in meters) covered by the waves is usually assimilated to the surface distance between the two recording sites. The PWV is calculated as PWV = L/Δt. The most commonly used method for estimating the transit time is the foot-to-foot method. The foot of the wave is defined at the end of diastole when the steep rise in the wavefront begins. The transit time is the time of travel of the foot of the wave over a known distance (Figure 1) [10].

The measurement of aortic stiffness using the PWV is used in most epidemiologic studies searching for a correlation between aortic stiffness and cardiovascular mortality. Aortic stiffness is an independent predictor factor of cardiovascular events and mortality [7]. The correlation between increased PWV and cardiovascular mortality has been studied in patients with essential hypertension, diabetes mellitus, and end-stage renal disease [11,12,13]. In addition, there are studies supporting the existence of this correlation in the general population [14]. For these reasons, it is widely established that aortic stiffness is a strong independent factor of cardiovascular outcomes [7].

## 3. Central Aortic Pressure (CAP) and Augmentation Index (AIx)

**Figure 2:**
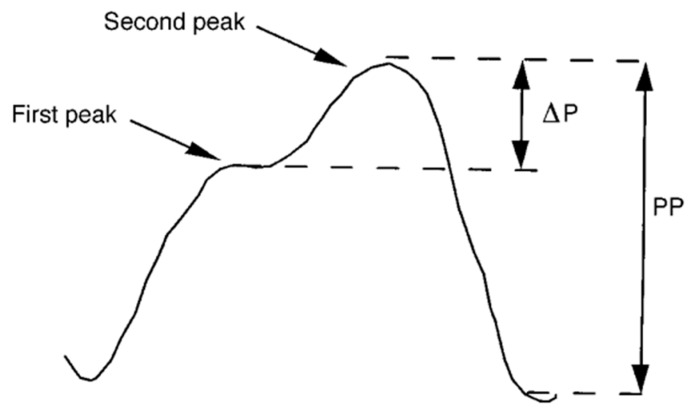
The augmentation index is defined as the difference between the second and first systolic peaks expressed as a percentage of the pulse pressure. ΔP: difference of pressure peaks; PP: pulse pressure [19].

The central aortic pressure (CAP) is an index of aortic stiffness, which was originally determined by complex and invasive aortic measurements [15]. The carotid pressure measured using carotid artery tonometry is an alternative valid, non-invasive, interchangeable method of CAP measurement, which is usually obtained transcutaneously at the right common carotid artery [16], while non-invasive central pressure estimations are accurate in patients with an abdominal aortic aneurysm, before and after endovascular repair [17]. O’Rourke et al., in their arterial waveform analysis, supported that the arterial pressure waveform is a composite of a forward-traveling wave, generated by left ventricular ejection, and a backward-traveling reflected wave arising from sites of impedance mismatch. This change in impedance is thought to generate numerous reflected ‘wavelets’ that sum together to produce a single ‘effective’ reflected wave, which is thought to augment or increase systolic pressure in the central arteries. The augmentation index (AIx) quantifies the extent of augmented pressure relative to the central pulse pressure [18]. The AIx is defined as the difference of pressures (ΔP = P2 − P1) between the pressure of the forward travelling wave (P1) and the pressure augmented by the reflected wave (P2) expressed as a percentage of the pulse pressure (PP). The AIx is calculated as AIx = ΔP/PP (Figure 2) [19].

The CAP and AIx hold a prognostic value of adverse cardiovascular events in various studies.Central hemodynamic indexes are independent predictors of future cardiovascular events and all-cause mortality [20]. The predictive value of the CAP and AIx for cardiovascular events has been studied in coronary artery disease patients, end-stage renal disease patients, and the general population [21,22,23].

## 4. Aortic Stiffness after EVAR

Bissacco et al., in their systematic review and meta-analysis, support the notion that EVAR and thoracic EVAR (TEVAR) lead to a significant increase in aortic stiffness with a respective increase in PWV compared to preoperative measurements [6]. This increase in PWV has been attributed to the increase in aortic rigidity that the implantation of aortic endoprostheses causes [6]. Similarly, Moulakakis et al., in their narrative review, support that the treatment of an AAA with EVAR reduces aortic compliance significantly, and an increase in arterial stiffness was uniformly observed in studies investigating patients following TEVAR, and the effect was more pronounced in young patients [24].Apart from a decrease in aortic compliance and an increase in aortic stiffness, EVAR has been related to reduced cardiac systolic function, heart diastolic dysfunction, and left ventricle hypertrophy [25,26]. Studies mention the possible harmful effect of EVAR not only on heart functions but also on other vascular beds, including kidney function [25,26,27].

In the literature, there are increasing numbers of studies that have investigated the properties of endografts, which are strongly related to the increase in aortic stiffness. In their study, Kadoglou et al. found that both the Dacron polyester and ePTFE endografts used in EVAR increased the PWV, while polyester-covered endografts caused even greater PWV elevation [28]. Similarly, in their study, Hori et al. revealed the idea that the selection of a specific device is related to increased PWV after TEVAR. Furthermore, Hori et al. found that the treatment length of the thoracic aorta and the length of the endograft are independent factors that are positively correlated with an increase in PWV after TEVAR, although the treatment site does not have an independent effect on the PWV [29]. On the contrary, in their study, Yamashita et al. found that the treatment length and device type were not predictors of an increase in the PWV after TEVAR [30].

## 5. Rationale of the Study

This study has a prospective, observational, monocentric, single-arm design, and it is intended to evaluate whether there is a correlation between the lengths of various endografts implanted during EVAR and an increase in the PWV, CAP, and AIx postoperatively compared to the preoperative values. To the best of our knowledge, this is the first study to investigate the correlation between endograft length and the postoperative increase in the PWV, CAP, and AIx in patients with AAAs subjected to EVAR.

Additionally, this study intends to determine other endograft properties related to increases in the PWV and aortic stiffness, such as the material of the endograft. Other endpoints to be studied include the existence of immediate postoperative myocardial and kidney injury after EVAR.

The aim of this study is to add new data to the existing knowledge and literature regarding the potentially harmful effect of endograft deployment during EVAR procedures on the cardiovascular system by investigating various properties of the endograft related to the increase in the PWV, CAP, and AIx. The prediction of cardiovascular events caused by endograft-related increased aortic stiffness could contribute to the improvement in various endograft properties so that the impact of endografts on the native aortic wall can be minimized.

## 6. Study Design

This is a prospective, observational, monocentric, single-arm study. Patients with AAAs fulfilling the inclusion criteria and managed with EVAR will be consecutively enrolled in our study. Informed written consent will be obtained from all the patients participating in our study. A thorough medical history of every patient will be obtained, including the patients’ demographics, past medical and surgical history, family medical history, comorbidities, medications, smoking habits, and alcohol consumption. Every patient will be subjected to a preoperative computed tomography angiogram (CTA) so that the anatomical characteristics of the AAA can be recorded. Digital subtraction angiography (DSA) images during the EVAR procedure will be collected. Details regarding the operation as well as any complications during the operation and the first 48 h postoperative period will be recorded. The PWV, CAP, and AIx will be measured preoperatively and postoperatively during the first 24 h after EVAR. A full blood count and biochemical and coagulation investigations will be ordered for all the patients preoperatively and at 6 h, 24 h, and 48 h postoperatively.

## 7. Sample Size

A power analysis was conducted around the primary endpoint regarding the effect of graft length on the PWV, CAP, and AIx. The expected effect was based on the study of Hori et al., where the specific measure is an independent prognostic factor [29]. The type I error was set at 0.05, while the minimum desired power equaled 0.8, based on the related literature. Considering a maximum of 10 confounders to be included in the model, a sample size equal to 107 emerged, and no dropouts were considered in this estimation. The number of patients to be enrolled in this study is 107, and any patient not fulfilling the inclusion criteria or with missing information will be excluded and an additional patient will be prospectively recruited.

## 8. Inclusion/Exclusion Criteria

The eligible patients included in our study will meet the following criteria: adult male or female patients suffering from infrarenal AAAs and managed electively using aortobiiliac endografts (with extension to the external iliac arteries if demanded) according to the ESVS guidelines (AAAs with a diameter of ≥5.5 cm in men and ≥5 cm in women; rapid AAA growth of ≥1 cm/year) [4] (Table 1).

The exclusion criteria are as follows: patients with ruptured, inflammatory, or mycotic AAAs; patients with juxtarenal or thoracoabdominal aortic aneurysms subjected to chimney EVAR (ChEVAR); fenestrated EVAR (FEVAR) or branched EVAR (BEVAR); patients with connective tissue disorders (Marfan syndrome, Ehlers–Danlos syndrome, Behcet disease, etc.) or end-stage renal disease; patients with severe atherosclerotic disease of the brachiocephalic and/or right common carotid and/or internal carotid and/or right common iliac and/or external iliac and/or right common femoral arteries with severe hemodynamic changes; patients who have been subjected to previous operations (open and/or endovascular) in these arteries; patients who have been subjected to previous repairs of the aorta (open and/or endovascular of either the ascending aorta and/or aortic arch and/or descending thoracic and/or abdominal aorta); and patients subjected to EVAR with aortouniiliac devices or straight-tube endografts (Table 1).

## 9. Definition of Variables

The primary endpoint of this study is the identification of the correlation between the lengths of various endografts implanted during EVAR and the increase in aortic stiffness estimated using the PWV, CAP, and AIx postoperatively compared to the preoperative measurement of these indices. The preoperative CTAs of patients will be studied using the 3mensio vascular workstation (Pie Medical Imaging BV, Maastricht, The Netherlands), defining the centerline of the aortic lumen [31] so that the distance between the lowest renal artery and the right common iliac bifurcation can be calculated. The length of the endograft will be calculated by studying the preoperative CTA analysis with the 3mensio vascular workstation (Pie Medical Imaging BV, Maastricht, The Netherlands), the digital subtraction angiographies (DSAs) performed during the EVAR procedure, and the radiopaque markers of the endografts. The type of endograft, which will be used in every patient, will be left up to the discretion of the surgeons according to the anatomical characteristics of the AAA and the endograft availability. The PWV, CAP, and AIx measurements will be conducted using the system Complior (Colson, Les Lilas, France), following the recommendations of the Artery Society, the European Society of Hypertension Working Group on Vascular Structure and Function, and the European Network for Noninvasive Investigation of Large Arteries [32].

The secondary endpoints of this study include the correlation of other endograft properties related to aortic stiffness with increases in the PWV, CAP, and AIx and the occurrence of immediate postoperative myocardial and kidney injury within 48 h of the EVAR procedure. The myocardial injury will be diagnosed using the blood serum levels of high-sensitivity troponin I (hs-cTnI) at 6 h, 24 h, and 48 h postoperatively using the pathway for the investigation of patients with isolated suspected acute coronary syndrome optimized for the ARCHITECT_STAT_ hs-cTnI assay [33]. The upper reference limit (99th centile) ranges from 24 to 30 pg/mL in healthy populations, with a sex-specific upper reference range of 36 pg/mL for men and 15 pg/mL for women [34]. In the case of elevated hs-cTnI, the patients will be assessed by a cardiologist. Acute kidney injury (AKI) will be diagnosed using the serum levels of creatinine, the urine output, and the calculated estimated glomerular filtration rate (eGFR) according to the CKD-EPI equation [35] following the KDIGO criteria (Table 2) [36]. In the case of acute kidney injury, the patients will be assessed by a nephrologist.

EVAR procedures will be performed electively in a fully equipped operating suite with a radiolucent table under the fluoroscopic guidance of a portable C-arm device. Vitamin K antagonists (VKAs) and new oral anticoagulants (NOACs) will be discontinued at least five days and two days, respectively, prior to EVAR to mitigate the risk of excessive bleeding. VKAs will be bridged during the peri-operative period with low-molecular-weight heparin (LMWH). The patients will be subjected to either general or regional anesthesia during EVAR. Surgical access will be gained after standard surgical exposure of the common femoral arteries with the use of surgical cut-down and arteriotomy. During the procedure, antibiotic prophylaxis and intravenous heparin (100 IU/kg body weight) will be given, and a second heparin dose will be given when the EVAR procedure exceeds 2 h of operative time. Routinely, on the ipsilateral side, a stiff 0.035 wire will be introduced up to the aortic arch. A pigtail angiographic catheter will be advanced from the contralateral side to a level proximal to the renal arteries using a retrograde femoral approach. A DSA will be performed using the pigtail catheter. The main body of the endograft will then be deployed. After marking the position of the hypogastric arteries with DSA, a catheter will be advanced through the contralateral gate in order to gain contralateral wire access. A second stiff 0.035 wire will be introduced up to the aortic arch from the contralateral side. The extension limbs will then be deployed. If applicable, the legs will be extended to the common iliac bifurcation, taking care to avoid compromising the hypogastric arteries. After deployment of the endograft, balloon angioplasty will be performed at the docking zone of the main body as well as along the iliac limbs. Additional procedures, such as embolization of the hypogastric vessels, are generally performed prior to EVAR. Completion angiography to verify the exclusion of the aneurysm with patent renal arteries and distal runoff will be performed. The subcutaneous tissues and skin will be closed in a standard fashion. The patients will be discharged with lifelong single antiplatelet therapy. The patients receiving oral anticoagulants prior to EVAR will be discharged on LMWH for a period of 10 days, followed by bridging therapy in VKA patients by initiating VKAs along with continuation of LMWH until the target INR is reached, while in the NOAC patients, LMWH will be discontinued thereafter and NOACs will be initiated immediately without bridging therapy.

## 10. Data Collection

The data recorded in spreadsheets will include the patient demographics; past medical and surgical history; family medical history; comorbidities; medications; smoking habits; alcohol consumption; anatomical characteristics of the AAA; length and other properties of the endograft implanted; PWV, CAP, and AIx measurements before and after the operation; operative details (the operation time, radiation time, radiation dosage, and contrast quantity used);overall outcome of the procedure; any adverse events/complications during the operation and postoperatively; and hs-cTnI, creatinine, urine output, and eGFR preoperatively and at 6 h, 24 h, and 48 h postoperatively.

## 11. Statistical Analysis

The means of central tendency and dispersion will be used to describe all of the scale variables after examining their distribution using the Shapiro–Wilk test and QQ plots. Frequencies and percentages will be used in cases with categorical variables. The coefficients of the correlations between the graft length and primary outcome indices will be examined, while the relationships between these indices and all the confounders will be examined in order to develop a robust regression model for the PWV, CAP, and AIx. The presence of AKI and MI will also be considered in the study context, and association statistics such as the Pearson chi-square and Fisher’s exact test will be used to assess their incidence depending on the medical history of the patients or other comorbidities. A logistic regression model will be developed for each patient, in which treatment and other AAA characteristics will also be considered. The indices of interest will be recorded at 6, 24, and 48 h postoperatively, and complications will also be recorded. The analysis will be carried out using SPSS v 28.0, and the significance will be set at 0.05 in all cases.

## 12. Limitations

The present study offers some strengths including its prospective design and focus on the correlation of endograft properties implanted during EVAR and the PWV, CAP, and AIx in a cohort of AAA patients. Although the correlation between the endograft length and an increase in the PWV, CAP, and AIx has already been studied in patients subjected to TEVAR, this correlation has not been studied in EVAR yet. To the best of our knowledge, this is the first study to investigate the correlation between the EVAR endograft length and the postoperative increase in the PWV, CAP, and AIx in patients with AAAs. The patients included in our study will be adequately homogenous as they will be subjected to EVAR with aortobiiliac endografts fulfilling specific inclusion criteria. On the other hand, the strict inclusion and exclusion criteria implemented in order to accumulate a relatively homogenous group of patients will exclude patients subjected to ChEVAR, FEVAR, or BEVAR, resulting in a relatively small sample size. Moreover, our study is a monocentric, single-arm observational study. While our power analysis suggests that the results will be reliable, generalization of the results should be avoided.

## 13. Future Directions

The association of aortic stiffness with various properties of ChEVAR, BEVAR, or FEVAR endografts implanted in patients during the management of complex AAAs and thoracic aortic aneurysms should be studied, as more and more patients with complex aneurysms are managed with endovascular techniques. Studies with a multi-center design and a broader group of patients should be conducted so that more generalizable and reliable results can be produced. Investigating various properties of the endografts related to the increase in aortic stiffness and predicting cardiovascular events caused by increased aortic stiffness related to endograft implantation could contribute to an improvement in various endograft properties so that the endograft impact on the native aortic wall could be minimized. Sultan et al.present the idea of an endoprosthesis that will retain the ability to expand in systole and collapse in diastole. After implantation, it would return the elastic recoil to the heart, creating an almost standard aortic flow curve [37]. Further simple, non-invasive, reliable, reproducible, affordable, and accessible indices assessing aortic stiffness should be studied and offered to medical professionals, as the need for deeper and wider investigations of hemodynamics and its role in adverse cardiovascular events still remains.

## 14. Conclusions

The PWV, CAP, and AIx increase significantly postoperatively after EVAR and TEVAR compared to preoperative measurements. This increase in the PWV, CAP, and AIx has been attributed to the increase in aortic rigidity caused by the implantation of aortic endoprostheses. Aortic stiffness has been established as an independent factor for increased cardiovascular disease risk. Several studies in the literature have investigated the properties of endografts, which are related to the increase in the PWV, CAP, and AIx, and have attempted to deduce the possible deleterious effects of endografts on cardiovascular events. This study will assess the correlation between the endograft length and the postoperative increase in the PWV, CAP, and AIx in patients with AAAs subjected to EVAR as well as respective correlations regarding other properties of the endograft. Moreover, acute myocardial and kidney injury after EVAR will be studied. The aim of this study is to contribute to the improvement of various endograft properties so that the impact of endografts on the native aortic wall can be minimized and, thus, cardiovascular events caused by endograft-related increases in aortic stiffness can be prevented.

## Figures and Tables

**Table 1:** Inclusion and exclusion criteria.

Inclusion Criteria	Exclusion Criteria
Adult male or female patients	Ruptured, inflammatory, or mycotic AAA
Infrarenal AAA ^1^	Juxtarenal or thoracoabdominal aortic aneurysms
AAA diameter of ≥5.5 cm in men or ≥5 cm in women or rapid AAA growth of ≥1 cm/year	ChEVAR ^3^, BEVAR ^4^, FEVAR ^5^ or EVAR with aortouniiliac devices or straight tube endografts
Elective EVAR ^2^	Connective tissue disorders
Aortobiiliac endografts	End-stage renal disease
Extension to external iliac arteries if demanded	Severe atherosclerotic disease with severe hemodynamic changes in arteries affecting PWV ^6^, CAP ^7^ and AIx ^8^ measurement
	Operations (open and/or endovascular) of arteries affecting PWV, CAP, AIx measurement
	Previous repairs of the aorta (open and/or endovascular)

^1^ Abdominal aortic aneurysm, ^2^ endovascular aortic repair, ^3^ chimney EVAR, ^4^ branched EVAR, ^5^ fenestrated EVAR, ^6^ pulse wave velocity, ^7^ central aortic pressure, ^8^ augmentation index.

**Table 2:** KDIGO criteria for acute kidney injury [36].

**AKI ^1^ Definition**		
1	Increase in sCr ^2^ ≥ 0.3 mg/dL (≥26.5 μmol/L) within 48 h; or	
2	Increase in sCr ≥ 1.5 times baseline, which is known or presumed to have occurred within the prior 7 days; or	
3	Urine volume <0.5 mL/kg/h for 6 h.	
**AKI Staging**		
Stage 1	1.5–1.9 times baseline OR ≥0.3 mg/dL (≥26.5 μmol/L) absolute increase in sCr	Urine volume < 0.5 mL/kg/h for 6–12 h
Stage 2	sCr ≥ 2.0–2.9 times baseline	Urine volume < 0.5 mL/kg/h for ≥12 h
Stage 3	Increase in sCr ≥ 4.0 mg/dL (≥353.6 μmol/L) OR Initiation of renal replacement therapy OR In patients < 18 years, decrease in eGFR ^3^ to <35 mL/min per 1.73 m^2^	Urine volume < 0.3 mL/kg/h for ≥24 h OR Anuria for ≥12 h

^1^ AKI: acute kidney injury; ^2^ sCr: serum creatinine; ^3^ eGFR: estimated glomerular filtration rate.

## Data Availability

This article represents a study protocol. The data will be generated in the course of the study and published later.
